# When chest pain and troponin elevation aren’t acute coronary syndrome: a cardiovascular case report of atypical pheochromocytoma

**DOI:** 10.1093/ehjcr/ytag544

**Published:** 2026-08-07

**Authors:** Fadoua Achour, Soukaina Cherkaoui, Jamila Zarzour, Mohamed Cherti

**Affiliations:** Department of Cardiology B, Ibn Sina University Hospital, Rue Lamfadel Cherkaoui, B.P. 6527, Rabat, Morocco; Department of Cardiology B, Ibn Sina University Hospital, Rue Lamfadel Cherkaoui, B.P. 6527, Rabat, Morocco; Department of Cardiology B, Ibn Sina University Hospital, Rue Lamfadel Cherkaoui, B.P. 6527, Rabat, Morocco; Department of Cardiology B, Ibn Sina University Hospital, Rue Lamfadel Cherkaoui, B.P. 6527, Rabat, Morocco

**Keywords:** Pheochromocytoma, Acute coronary syndrome, MINOCA, Myocardial injury, Case report, Secondary hypertension

## Abstract

**Background:**

Pheochromocytoma is a rare catecholamine-secreting tumour with diverse cardiovascular manifestations. Presentation with chest pain and troponin elevation may closely mimic acute coronary syndrome (ACS), potentially leading to diagnostic delay.

**Case summary:**

We report the case of a 44-year-old woman who presented to the emergency department with acute chest pain and elevated cardiac biomarkers. Electrocardiography showed sinus rhythm with diffuse T-wave inversions, and high-sensitivity troponin levels were markedly elevated, leading to an initial diagnosis of non-ST-segment elevation myocardial infarction. Urgent coronary angiography, however, revealed angiographically normal coronary arteries. Cardiac magnetic resonance imaging demonstrated regional wall motion abnormalities without myocardial oedema or late gadolinium enhancement, excluding myocarditis and Takotsubo cardiomyopathy and suggesting a non-ischaemic mechanism of myocardial injury. The association of paroxysmal hypertension and adrenergic symptoms prompted further investigation, revealing markedly elevated plasma metanephrines, while abdominal imaging identified a left adrenal mass consistent with pheochromocytoma. Following adequate preoperative alpha- and beta-adrenergic blockade, the patient underwent successful surgical resection, resulting in complete clinical and cardiovascular recovery.

**Discussion:**

This case highlights the diagnostic challenge posed by pheochromocytoma presenting as ACS in the absence of obstructive coronary artery disease. It emphasizes the importance of reconsidering alternative diagnoses in patients with myocardial injury and normal coronary angiography, particularly when adrenergic symptoms are present.

Learning points

- Pheochromocytoma may mimic acute coronary syndrome with chest pain, troponin elevation, and ECG changes despite normal coronary arteries.
- Adrenergic symptoms in patients with MINOCA should prompt evaluation for catecholamine-secreting tumours.
- Surgical resection can lead to complete reversal of cardiovascular manifestations.

## Introduction

Chest pain associated with elevated cardiac troponin levels usually raises strong suspicion for acute coronary syndrome (ACS). However, myocardial injury may occur in the absence of obstructive coronary artery disease, a condition increasingly recognized as myocardial infarction with non-obstructive coronary arteries (MINOCA).^1^

Pheochromocytoma, a rare catecholamine-producing tumour, can closely mimic ACS, with presentations ranging from silent hypertension to life-threatening cardiovascular events.^2^

Excess catecholamines can induce myocardial injury through multiple mechanisms, leading to electrocardiographic changes and troponin elevation that resemble ischaemic heart disease.^2^

This case illustrates a pheochromocytoma presenting with myocardial injury initially misdiagnosed as ACS, highlighting the importance of reconsidering the diagnosis when clinical findings and test results are inconsistent.

## Summary figure

**Figure:**
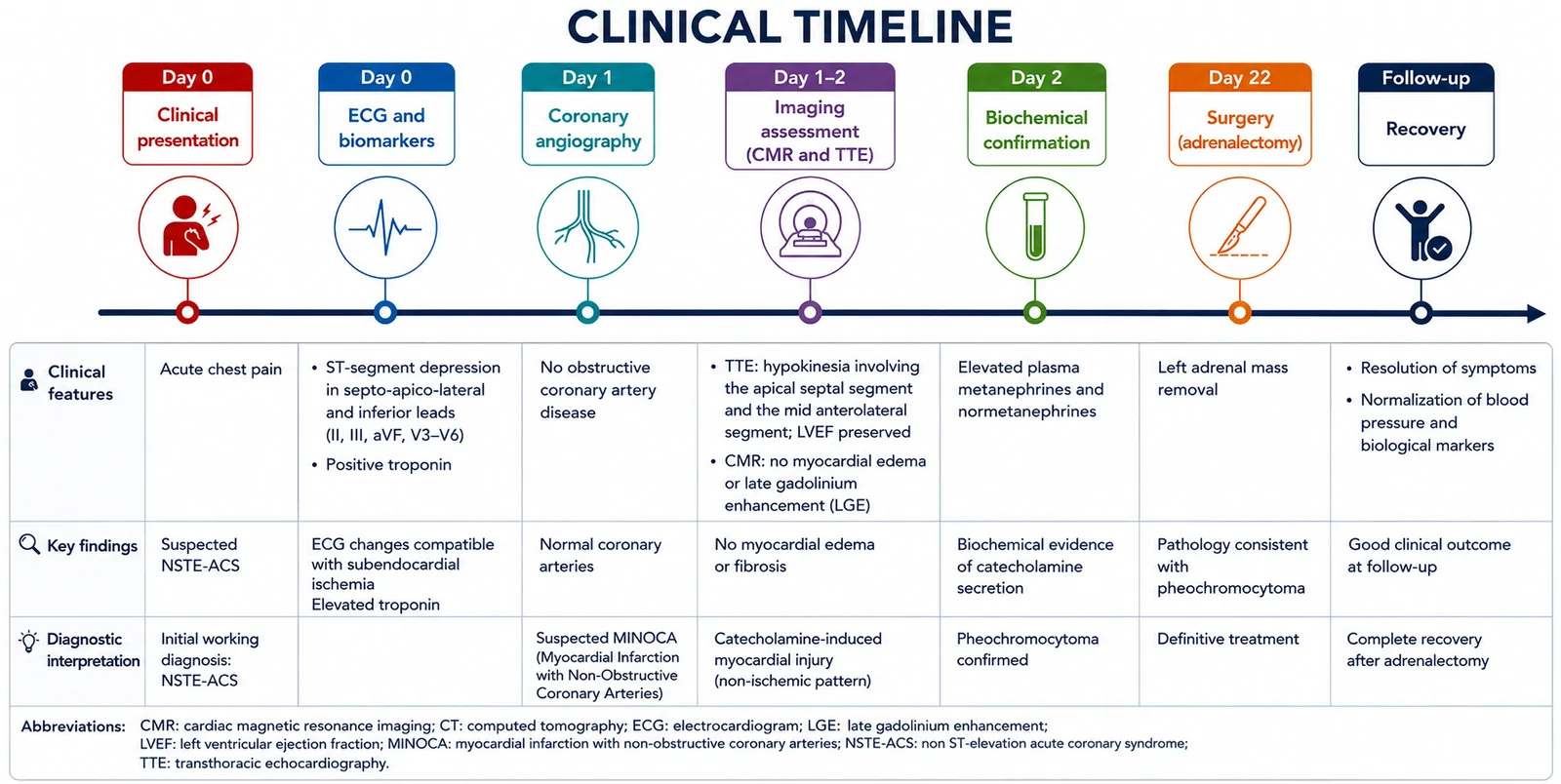


## Case Presentation

We report the case of a 44-year-old woman who presented to the emergency department with sudden-onset chest pain. Her cardiovascular risk factors included type 2 diabetes mellitus, diagnosed 5 years earlier, with mild diabetic retinopathy and nephropathy, as well as dyslipidemia.

The chest pain was retrosternal, oppressive, and prolonged, without radiation. It occurred in brief but intense episodes, typically during periods of marked hypertension. These paroxysms were consistently accompanied by profuse sweating and severe headaches. Between crises, the patient remained asymptomatic and normotensive.

On initial assessment, she was haemodynamically stable. Blood pressure was within the normal range, and cardiovascular examination was unremarkable. Blood pressure readings were symmetrical between limbs. Peripheral pulses were present and equal. Pulmonary auscultation was normal. The rest of the physical examination was normal.

Given the characteristics of the chest pain, an ECG and a chest X-ray were performed. The ECG revealed sinus tachycardia with electrical left ventricular hypertrophy and diffuse T-wave inversions affecting the septal, apicolateral, and inferior leads, while the chest X-ray was unremarkable (see Supplementary material online, *Figure S1*).

**Figure 1:**
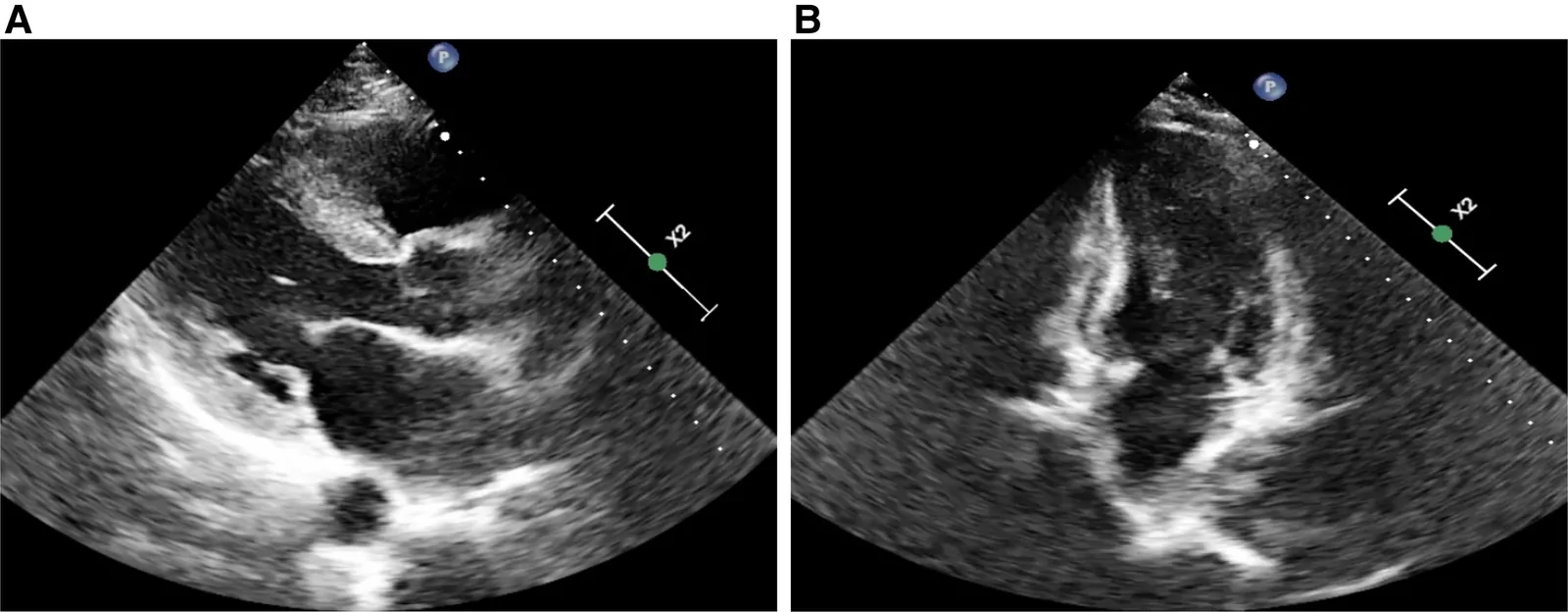
Transthoracic echocardiography demonstrating subtle hypokinesia of the apical septal and mid anterolateral segments, in the setting of mild left ventricular hypertrophy with preserved global systolic function. (*A*) Parasternal long-axis view. (*B*) Apical two-chamber view.

Cardiac biomarkers were elevated. High-sensitivity cardiac troponin demonstrated a dynamic rise, increasing from 0.17 ng/mL at baseline to 0.53 ng/mL at 1 h. Transthoracic echocardiography showed subtle regional wall motion abnormalities involving the apical septal segment and the mid anterolateral segment, in the setting of mild left ventricular hypertrophy and preserved global systolic function (*Figure 1*, Supplementary material online, *Videos S1*, *S2*, and *S3*).

**Figure 2:**
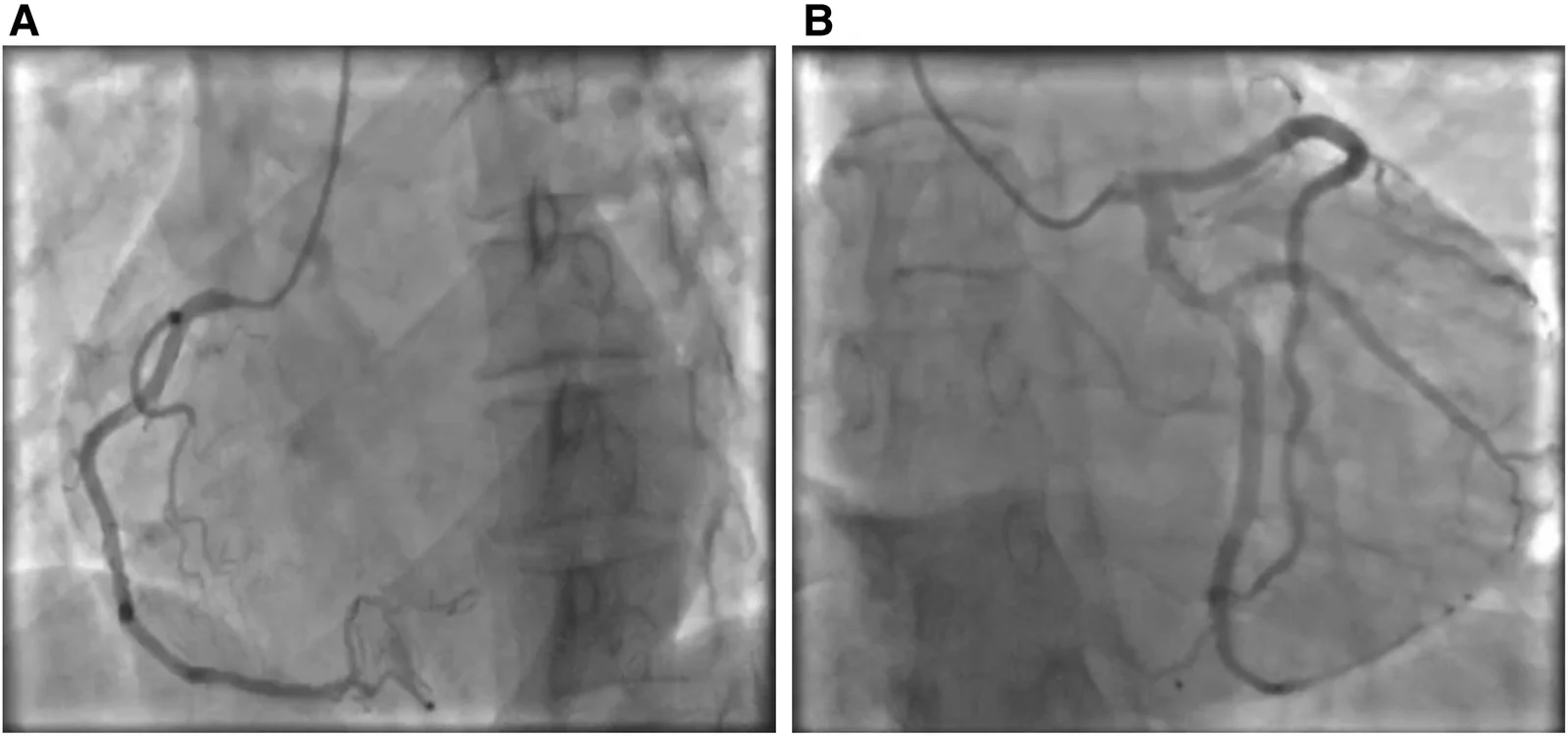
Coronary angiography demonstrating patent coronary arteries without significant stenosis. (*A*) Left coronary system without significant stenosis. (*B*) Right coronary artery without obstructive lesions.

Taken together, these findings raised suspicion for an acute coronary syndrome, and the patient underwent urgent coronary angiography. The coronary arteries were angiographically normal, with no evidence of obstructive disease (*Figure 2*).

In the absence of obstructive coronary disease, alternative diagnoses including MINOCA, myocarditis, and stress-induced cardiomyopathy were considered.

**Figure 3:**
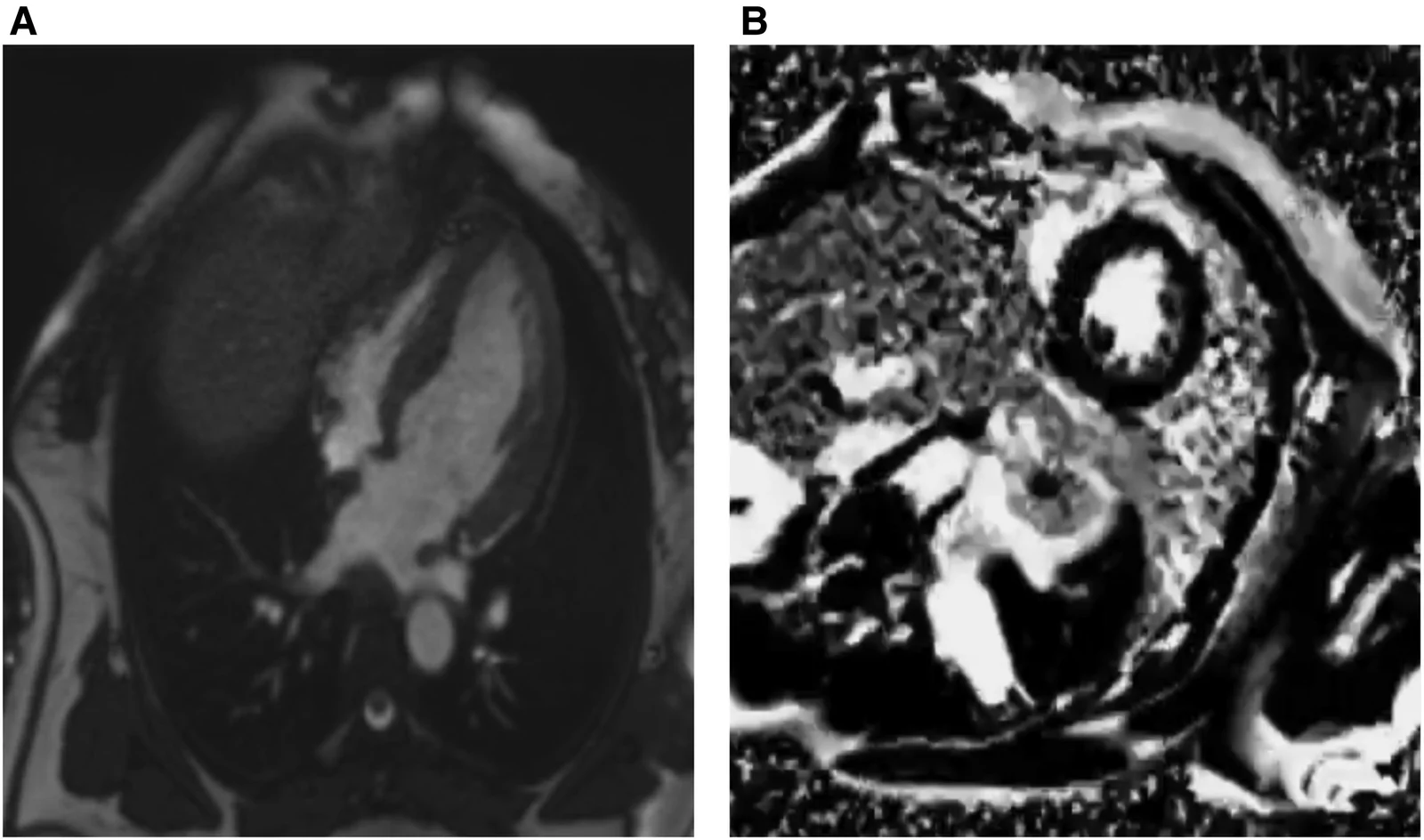
CMR demonstrating mild global and regional hypokinesia with preserved left ventricular ejection fraction, without evidence of myocardial oedema or late gadolinium enhancement. (*A*) Cine four-chamber view showing regional wall motion abnormalities. (*B*) LGE imaging showing no evidence of myocardial fibrosis or scar.

Cardiac magnetic resonance imaging (CMR) demonstrated mild global hypokinesia with regional hypokinesia involving the mid inferolateral segment and part of the mid anterolateral segment, as well as delayed contraction of the apical septal segment, in the setting of left ventricular hypertrophy consistent with hypertensive cardiomyopathy and preserved left ventricular ejection fraction. T2-weighted sequences showed no myocardial oedema, and no late gadolinium enhancement (LGE) was detected, without imaging features suggestive of myocarditis or typical Takotsubo syndrome. These findings supported a catecholamine-mediated myocardial injury in the absence of obstructive coronary artery disease (*Figure 3*, Supplementary material online, *Videos S4*, *S5*, and *S6*), and favouring a non-ischaemic mechanism of myocardial injury in the absence of obstructive coronary artery disease.

Considering the recurrent hypertensive episodes, associated adrenergic symptoms, and the absence of obstructive coronary disease, a catecholamine-secreting tumour was suspected. A workup for secondary hypertension was initiated.

Serum sodium, potassium, and chloride levels were all within normal limits, helping to rule out primary hyperaldosteronism and hypercortisolism. Renal function was preserved, but the urinary albumin-to-creatinine ratio was elevated, consistent with early-stage diabetic nephropathy. Long-term glycemic control was poor, with an HbA1c of 12%. Thyroid function tests were normal, excluding a thyroid-related aetiology.

**Figure 4:**
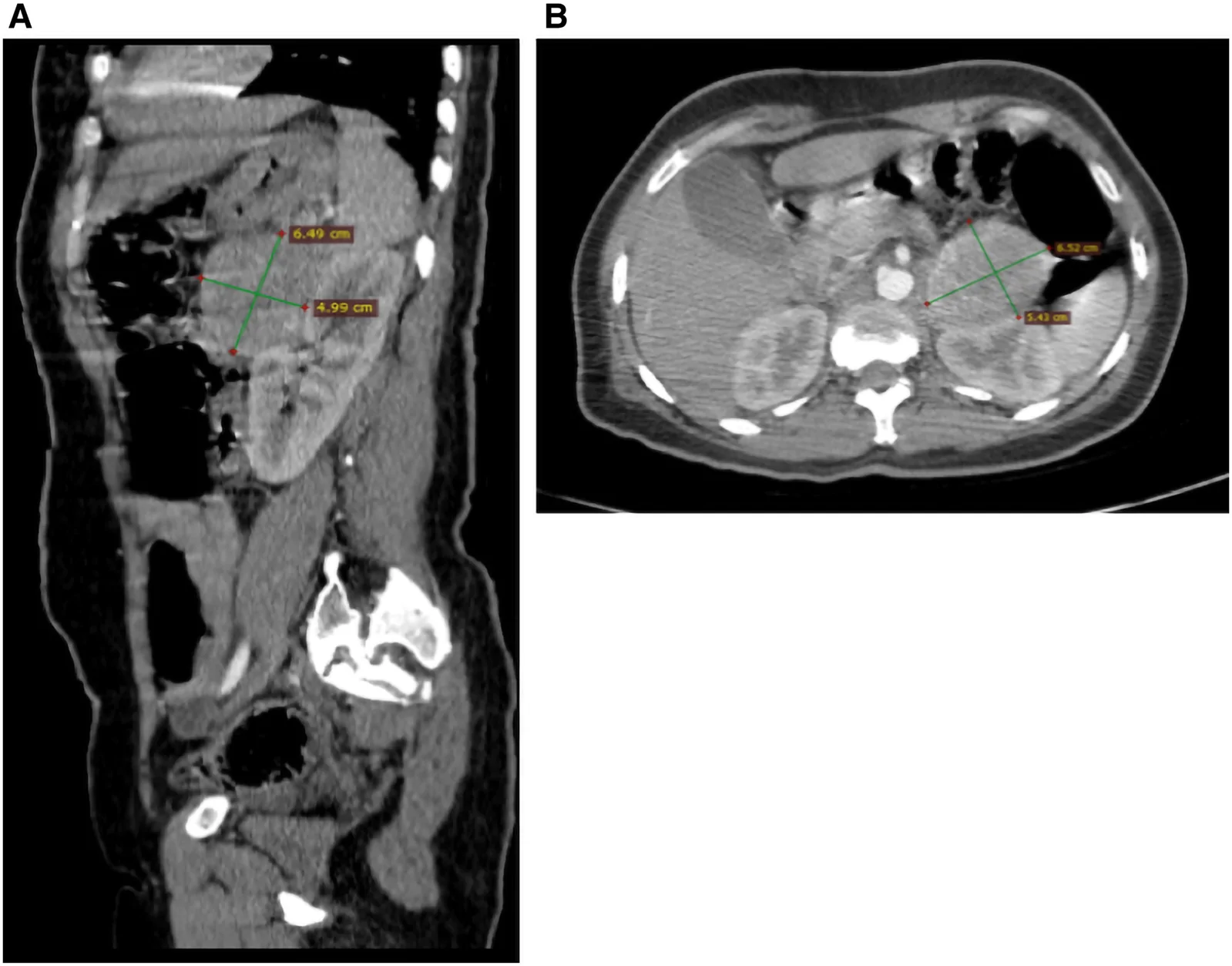
Abdominal computed tomography demonstrating a left adrenal mass. (*A*) Sagittal view. (*B*) Axial view showing a well-defined heterogeneous left adrenal tumour measuring approximately 65 × 50 × 66 mm, suggestive of pheochromocytoma.

To investigate the suspected adrenal origin, an abdominal CT scan was performed (*Figure 4*). It showed a well-defined mass in the left adrenal gland, measuring 65 × 50 × 66 mm, suggestive of pheochromocytoma.

Biochemical confirmation was obtained by measuring plasma-free metanephrines and normetanephrines under strictly controlled pre-analytical conditions. The levels were significantly elevated: Plasma metanephrine at 1 250 pg/mL (normal < 65 pg/mL) and normetanephrine at 3 980 pg/mL (normal < 196 pg/mL), confirming the diagnosis.

Screening for hereditary syndromes was indicated in the context of confirmed pheochromocytoma in a relatively young patient. Clinical evaluation showed no mucosal neuromas, marfanoid features, spinal pigmentation, or thyroid abnormalities. Laboratory tests, including serum calcium, phosphate, parathyroid hormone, and calcitonin, were within normal limits. The absence of clinical and biochemical abnormalities did not support an underlying genetic syndrome such as multiple endocrine neoplasia types 1, 2A, or 2B.

Preoperative medical preparation began with alpha-adrenergic blockade using doxazosin, starting at 0.5 mg/day and gradually titrated up to 6 mg/day. On the fourth day, beta-blockade was added with propranolol, initiated at 10 mg (¼ tablet of 40 mg) three times daily, and titrated up to 100 mg/day. The entire preoperative preparation period lasted 22 days, and she was referred to the urology department for surgical management.

**Figure 5:**
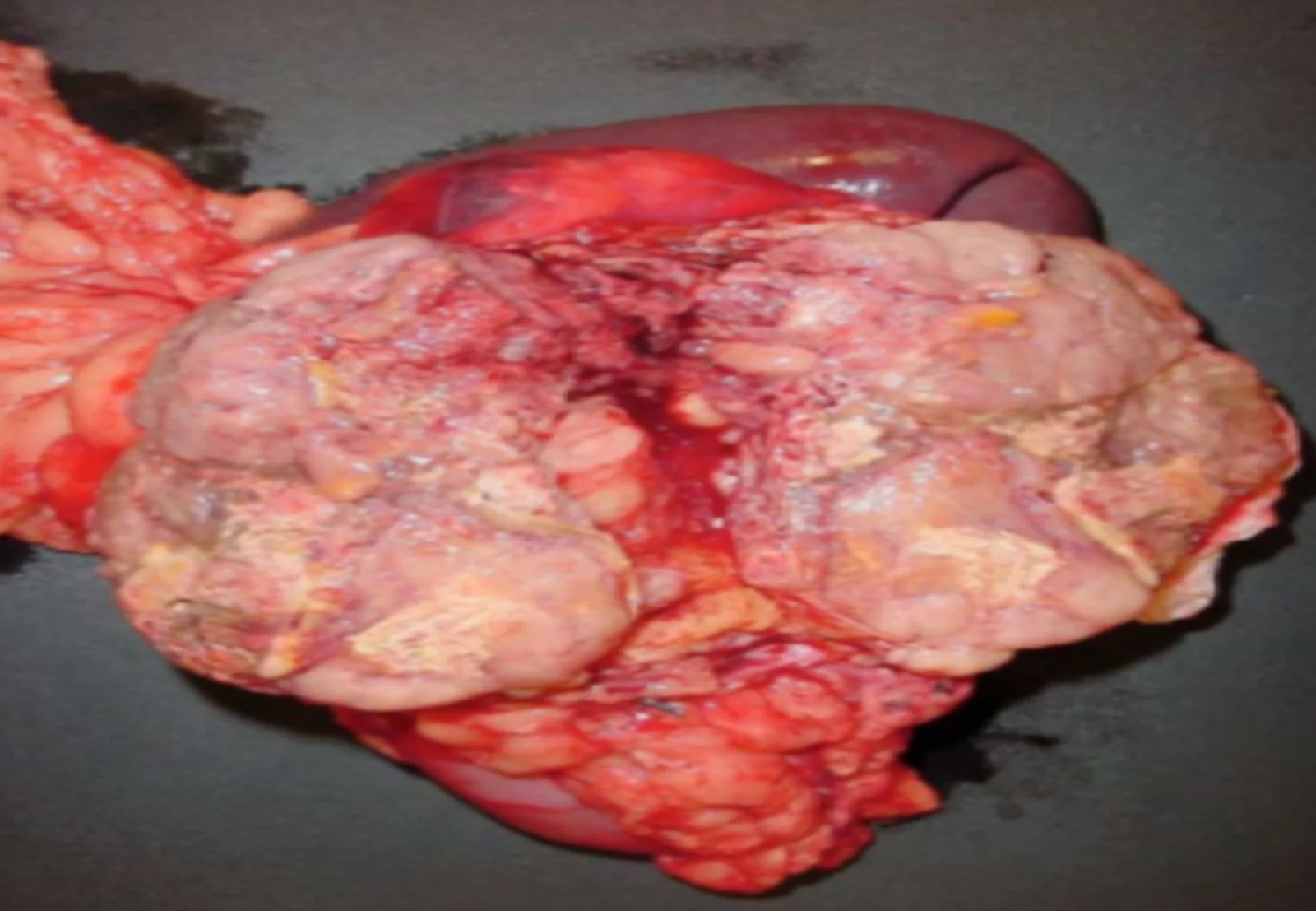
Gross specimen of the left adrenal mass following laparoscopic adrenalectomy.

The patient underwent a left laparoscopic adrenalectomy with the operative specimen subsequently analysed histologically, confirming the diagnosis (*Figure 5*). Blood pressure was monitored intraoperatively, and hypertensive crises during tumour manipulation (up to 250/130 mmHg) were managed effectively with deep anaesthesia and intravenous nicardipine. Postoperative care included 2 days in the intensive care unit before transfer to the surgical ward.

The postoperative evolution was overall favourable. Blood pressure normalized without antihypertensive medication, and adrenergic symptoms resolved completely, as confirmed during follow-up consultations. Sinus tachycardia persisted transiently but improved after correction of mild anaemia.

Hydrocortisone therapy was started for transient adrenal insufficiency, later discontinued after adrenal recovery. At follow-up, plasma metanephrine and normetanephrine levels were within the normal range, and imaging showed no signs of recurrence, supporting a complete biochemical and radiological remission.

At follow-up, electrocardiography demonstrated complete normalization of previously observed T-wave inversions (see Supplementary material online, *Figure S2*). Repeat transthoracic echocardiography showed complete resolution of regional wall motion abnormalities, including normalization of lateral wall contractility, with preserved left ventricular systolic function (see Supplementary material online, *Video S7*).

These findings strongly support a reversible catecholamine-mediated myocardial dysfunction related to pheochromocytoma.

The patient demonstrated excellent adherence to the medical regimen and postoperative monitoring, reporting no significant side effects from either the preoperative alpha- or beta-blockade.

Long-term follow-up was planned with measurement of plasma or urinary methoxy derivatives (metanephrines and normetanephrines) at 3 and 6 months postoperatively, along with abdominal computed tomography at 6 months to reassess the adrenal region and exclude recurrence.

## Discussion

Pheochromocytoma is a rare catecholamine-secreting tumour arising from the adrenal medulla or, less frequently, extra-adrenal paraganglia, first described by Frankel in 1886. Despite medical advances, its diagnosis remains challenging due to its variable clinical presentation and low prevalence, estimated between 0.1% and 0.6% among hypertensive patients, with many cases discovered only postmortem.^3^

While the classic triad of paroxysmal hypertension, headaches, and sweating is well known, many patients present with atypical symptoms such as palpitations, anxiety, or abdominal pain, and some remain normotensive between episodes.^2,4,5^ This protean clinical presentation has earned pheochromocytoma the label of a ‘clinical chameleon,’ often leading to misdiagnoses such as panic disorder, migraine, or ACS.

Cardiac involvement may be the presenting feature of pheochromocytoma, occasionally mimicking MINOCA or Takotsubo syndrome, which remains a diagnosis of exclusion according to the International Takotsubo Diagnostic Criteria and requires exclusion of pheochromocytoma and other catecholamine-secreting tumours.^4^ In this context, catecholamine excess represents a key alternative mechanism of myocardial injury that must be considered. In our case, initial features suggested ACS, but the absence of coronary obstruction prompted further evaluation, leading to the diagnosis of pheochromocytoma. Catecholamine excess may reproduce several hallmarks of ACS, including chest pain, dynamic troponin elevation, ECG repolarization abnormalities, and transient regional wall motion abnormalities, even in the absence of epicardial coronary obstruction. This overlap frequently creates a diagnostic challenge and may initially lead clinicians towards an ischaemic aetiology.

This case highlights the value of multimodal imaging and repeated diagnostic reassessment when coronary angiography is normal despite biochemical evidence of myocardial injury. However, adrenergic signs, such as episodic hypertension and sweating, were initially underappreciated.

ECG abnormalities in pheochromocytoma-related cardiac injury are non-specific and may include transient ST-segment changes, T-wave inversions, Q waves, QT prolongation, and arrhythmias such as atrial fibrillation or ventricular tachycardia. These changes are typically transient and may regress after tumour resection.^6^

The pathophysiology of catecholamine-induced cardiomyopathy remains incompletely understood and is likely multifactorial. Excess catecholamine exposure induces myocardial injury through β-adrenergic overstimulation with intracellular calcium overload, coronary vasoconstriction and microvascular dysfunction resulting in functional ischaemia, and direct catecholamine-mediated cardiotoxicity via oxidative stress and mitochondrial dysfunction. Together, these mechanisms lead to reversible myocardial stunning and transient systolic dysfunction, frequently in the absence of obstructive coronary artery disease.^6,7^ This is in keeping with the reversible catecholamine-mediated myocardial involvement observed in our patient.

Pheochromocytoma should be suspected in patients with unexplained myocardial injury, particularly when associated with paroxysmal hypertension or signs of sympathetic overactivity. Plasma-free metanephrines and normetanephrines are the most sensitive biomarkers and should be measured under appropriate conditions.^6,8^ In our patient, markedly elevated levels confirmed the diagnosis.

Imaging plays a key role in both detection and characterization. CT is the first-line modality, offering excellent anatomical detail. MRI offers superior soft tissue contrast, particularly in extra-adrenal locations, while functional imaging, such as MIBG scintigraphy or PET, may help detect multifocal, metastatic, or hereditary forms.^6,8,9^

Surgical resection is the definitive treatment but should only be performed after adequate alpha-adrenergic blockade. Beta-blockers are introduced afterwards to control reflex tachycardia and avoid unopposed alpha stimulation, which could trigger hypertensive crises. Calcium channel blockers may be added in cases of resistant hypertension or poor alpha-blocker tolerance.^7,8^

The perioperative period requires close haemodynamic monitoring, as tumour manipulation may provoke acute catecholamine release and precipitate hypertensive crises.^8^ With adequate preoperative preparation and experienced anaesthetic management, laparoscopic adrenalectomy is generally safe and well tolerated.^7,8^ Postoperatively, most patients experience rapid clinical improvement, including normalization of blood pressure and resolution of catecholamine-related symptoms.^8^ In patients with cardiac involvement, ventricular function often recovers after tumour removal, although prolonged exposure to catecholamine excess may be associated with persistent myocardial fibrosis in some cases.^7,10^

This case underscores the importance of early recognition, careful diagnostic reasoning, and coordinated multidisciplinary care to prevent irreversible cardiovascular complications.

Although the initial presentation fulfilled the working diagnosis of MINOCA, the final diagnosis was catecholamine-mediated myocardial injury secondary to pheochromocytoma.

## Conclusion

Pheochromocytoma should be considered in patients presenting with chest pain, troponin elevation, and non-obstructive coronary arteries, particularly when adrenergic symptoms are present. Early recognition and multidisciplinary management are essential, as timely surgical resection can prevent life-threatening complications and allow complete cardiovascular recovery.

## Supplementary Material

ytag544_Supplementary_Data

## Data Availability

All relevant data are included within the article.

## Lead author biography

I am a cardiology resident at Ibn Sina University Hospital in Rabat, Morocco, with a particular interest in cardiovascular imaging and atypical cardiovascular presentations. I am especially interested in cases that remind us how important careful clinical assessment and multimodality imaging can be in everyday practice.

## Notes

**Consent:** Written informed consent was obtained from the patient for publication of this case report and accompanying images. A copy of the written consent is available for review by the Editor-in-Chief of this journal upon request.
