# Supplementary material for: When chest pain and troponin elevation aren’t acute coronary syndrome: a cardiovascular case report of atypical pheochromocytoma
Source: Eur Heart J Case Rep. 2026 Aug 7;10(8):ytag544. doi: 10.1093/ehjcr/ytag544 (PMC13449602; doi:10.1093/ehjcr/ytag544)
Supplement: Supplementary file 1 [file ytag544_supplementary_data.zip › Supplementary Material.docx]

**Supplementary Material**

**Supplementary Figure S1.** Electrocardiogram demonstrating sinus rhythm with diffuse T-wave inversions in the septal, apicolateral, and inferior leads.

**Supplementary Figure S2.** Follow-up electrocardiogram demonstrating normalization of the previously observed T-wave abnormalities.

**Supplementary Video 1.** Transthoracic echocardiography, parasternal long-axis view, demonstrating preserved global systolic function.

**Supplementary Video 2.** Transthoracic echocardiography, apical two-chamber view, demonstrating preserved global systolic function.

**Supplementary Video 3.** Transthoracic echocardiography (apical four-chamber view) demonstrating subtle hypokinesia of the apical septal and mid-anterolateral segments.

**Supplementary Video 4.** Cine CMR four-chamber view demonstrating delayed and reduced contraction of the apical septal segment.

**Supplementary Video 5.** Cine CMR short-axis view demonstrating mild global hypokinesia with regional hypokinesia involving the mid inferolateral segment and part of the mid-anterolateral segment.

**Supplementary Video 6.** T2-STIR cardiac magnetic resonance short-axis sequence showing no definite myocardial edema.

**Supplementary Video 7.** Follow-up transthoracic echocardiography demonstrating complete resolution of regional wall motion abnormalities.
